# Forward Dynamics: Modern Insights into Mitral Systolic Anterior Motion

**DOI:** 10.3390/jcdd13080397

**Published:** 2026-08-19

**Authors:** Fatima Zahra Samet Bouhaik, Ilenia Monaco, Mounia Sedrati, Alix Bouvet, Benedicte Gervais, Valeria Trivelloni, Yassine Bencharef, Fouad Mohammed Sekkal, Dario Bottigliero

**Affiliations:** 1 Department of Cardiology, Centre Hospitalier General Victor Jousselin de Dreux, 28100 Dreux, France; 2 Emergency Department, Centre Hospitalier Régional et Universitaire de Tours, 37170 Tours, France

**Keywords:** systolic anterior motion, hypertrophic cardiomyopathy, left ventricular outflow tract obstruction, mitral valve, septal myectomy, mitral regurgitation, mavacamten, alcohol septal ablation, surgical management, echocardiography

## Abstract

Systolic anterior motion (SAM) of the mitral valve can occur either in association with or in the absence of hypertrophic obstructive cardiomyopathy (HOCM). SAM induces dynamic left ventricular outflow tract obstruction (LVOTO) and, in the majority of cases, is associated with a substantial degree of mitral regurgitation (MR) that significantly impacts patient morbidity and mortality. This narrative review explores the contemporary understanding of the pathophysiology, diagnosis, and management of SAM, focusing particularly on surgical strategies and the novel therapeutic class of cardiac myosin inhibitors. Extended septal myectomy remains the gold-standard treatment for HOCM-related SAM, yielding superior long-term outcomes compared to alcohol septal ablation (ASA). Advanced imaging modalities, including three-dimensional transesophageal echocardiography (3D-TEE), enable precise pre-operative characterization of the mitral valve apparatus. Some patients may benefit from septal reduction strategies while concomitant mitral valve interventions are generally reserved for highly selected cases with intrinsic valve pathology or persistent residual SAM, thereby avoiding unnecessary valvular manipulation and its potential hemodynamic risks. Mavacamten, a selective cardiac myosin inhibitor, represents an important advance in pharmacological management, achieving a mean LVOT gradient reduction of 37.2 mmHg in symptomatic patients. Furthermore, data from the MARVEL registry confirm the real-world clinical efficacy of mavacamten in obstructive hypertrophic cardiomyopathy, with 86% of patients successfully down-staged to NYHA functional class I–II. Although ASA serves as a viable alternative to surgery, it entails a higher risk of conduction abnormalities requiring permanent pacemaker implantation and subsequent re-intervention. Beyond classical hypertrophic SAM, this review addresses the diagnosis and management of post-mitral repair complications and non-hypertrophic variants. Optimal management and risk stratification remain an evolving field requiring a multidisciplinary Heart Team approach, leverage of advanced imaging, and adoption of novel medical therapies. Interventional strategies must be carefully tailored to maximize the efficacy-to-safety profile on an individualized patient basis.

## 1. Introduction

Systolic anterior motion (SAM) of the mitral valve induces dynamic obstruction of the left ventricular outflow tract (LVOTO) and precipitates secondary mitral regurgitation (MR). While SAM is classically identified as the hallmark of hypertrophic obstructive cardiomyopathy (HOCM), it is increasingly recognized in a spectrum of non-hypertrophic conditions, including phenotypes completely devoid of left ventricular hypertrophy. Driven by innovations in diagnostic imaging and targeted surgical/pharmacological interventions, the clinical characterization of SAM has significantly matured [1]. Severe LVOTO secondary to SAM provokes debilitating symptoms, accelerates heart failure progression, triggers ventricular arrhythmias, and elevates the risk of sudden cardiac death (SCD) in affected patient populations. Consequently, a deeper understanding of the multifactorial etiologies and mechanistic drivers of SAM is paramount for optimizing patient care and refining long-term prognostic models [2].

## 2. Methods

### 2.1. Literature Search Strategy and Article Selection

To provide a comprehensive synthesis of contemporary insights into systolic anterior motion (SAM) of the mitral valve, a broad literature search was conducted across PubMed, EMBASE, Web of Science, and Google Scholar. The search strategy targeted English-language studies published through recent years, utilizing combinations of key search terms including the following: systolic anterior motion, SAM, hypertrophic cardiomyopathy, septal myectomy, alcohol septal ablation, mavacamten, mitral valve repair, and left ventricular outflow tract obstruction.

### 2.2. Synthesis and Selection Strategy

As this is a qualitative narrative review, the literature was curated based on thematic relevance, clinical importance, and contributions to understanding the evolving diagnostic and therapeutic landscapes of SAM. Primary consideration was given to seminal clinical trials, large observational registries, landmark surgical and percutaneous cohort studies, and modern imaging advancements. A total of 58 representative publications were selected to cover the spectrum of SAM, encompassing both classic hypertrophic states and non-hypertrophic or post-procedural variants.

## 3. Pathophysiology of Systolic Anterior Motion

### 3.1. SAM in Hypertrophic Cardiomyopathy

The pathophysiology of SAM in HOCM is inherently multifactorial, arising from a complex interplay of anatomical and hemodynamic determinants rather than a single structural defect [2]. Although asymmetric septal hypertrophy (ASH) and mechanical narrowing of the LVOT are cardinal features, septal thickness alone does not reliably predict the severity of dynamic obstruction. Instead, several phenotypic geometric anomalies are implicated in the initiation of SAM, including: (1) elongated anterior mitral leaflets, (2) redundant or thickened posterior leaflets, (3) anterior or inward displacement of the papillary muscles, and (4) an altered ratio between leaflet lengths and LVOT cross-sectional dimensions. Mid-to-late systolic anterior displacement of the mitral leaflets into the outflow tract represents the definitive mechanism of obstruction. This phenomenon is driven by flow acceleration within the narrowed LVOT, which generates a Venturi effect alongside high hydrodynamic drag forces acting directly on the redundant leaflet tissue. This distortion perturbs normal systolic coaptation, resulting in severe secondary MR characterized by an eccentric, posteriorly directed jet [3].

Hypertrophic cardiomyopathy (HCM) frequently manifests in a subclinical form before progressing to manifest hypertrophy. This pre-hypertrophic stage enables the identification of pre-symptomatic pediatric patients. Dyskinesis of the aorto-mitral apparatus combined with anterior leaflet elongation represents a characteristic phenotype in these children, placing them at heightened risk for developing SAM. Furthermore, the burden of myocardial fibrosis dictates the degree of excursion of the aorto-mitral structures, contributing to the fibrotic remodeling that predisposes to LVOTO [4].

### 3.2. SAM Without Left Ventricular Hypertrophy

Transcatheter aortic valve replacement (TAVR) presents distinct challenges, carries an inherent risk of inducing unmasked or latent SAM, and demands rigorous echocardiographic monitoring. Reversible SAM precipitated by myocardial injury has also been characterized following acute myocardial infarction. The underlying mechanism involves apical akinesia accompanied by compensatory hyperkinesis of the basal segments, which alters left ventricular configuration and flow vector dynamics, inducing outflow tract obstruction in the absence of pre-existing hypertrophy. Fortunately, optimal medical management—specifically combining beta-blockade, afterload adjustment, and meticulous volume stabilization—frequently achieves rapid reversal of SAM within weeks. Thus, SAM can manifest abruptly during acute hemodynamic shifts, independent of fixed anatomical abnormalities [5].

Intraoperative SAM has been documented following surgical aortic valve replacement (AVR) in adults presenting with severe aortic stenosis and concentric hypertrophy. Acute relief of chronic afterload in a patient with concentric left ventricular hypertrophy and a narrow aortic annulus can trigger marked SAM, dynamic LVOTO, and severe hemodynamic collapse—a phenomenon termed the “suicide left ventricle”. This rare but catastrophic condition occurs when a small baseline outflow tract is further compromised by surrounding septal mass. Additionally, non-hypertrophic SAM can be caused by congenital anomalies, such as anomalous chordae tendineae associated with atrioventricular septal defects [6].

## 4. Distinct SAM Phenotypes and Their Mechanisms

### 4.1. SAM Heterogeneity and Clinical Significance

Systolic anterior motion (SAM) of the mitral valve represents one of the most complex and heterogeneous phenomena in cardiology, extending far beyond its classic association with hypertrophic cardiomyopathy (HCM) [7]. The recognition that SAM can arise from fundamentally different anatomical, hemodynamic, and pathophysiological mechanisms has important diagnostic and therapeutic implications. Given the rarity and marked heterogeneity of some clinical phenotypes, robust large-cohort studies in the literature remain scarce; consequently, available evidence is heavily drawn from case reports and small observational series, which nonetheless provide valuable insights into managing these complex anatomical and hemodynamic presentations [8]. Recent advances in multimodality imaging and hemodynamic understanding have revealed that SAM phenotypes warrant distinct classification systems, as each subtype requires tailored diagnostic approaches and management strategies that may be diametrically opposed.

The traditional paradigm emphasized septal hypertrophy as the primary driver of LVOT obstruction through SAM, but contemporary investigations have revealed that anatomically predisposed leaflets interact with altered ventricular geometry and flow dynamics in complex ways [8]. Understanding the mechanistic basis of each SAM phenotype is essential for optimizing clinical outcomes, particularly when conventional therapeutic approaches may worsen hemodynamics or lead to incomplete treatment. The main phenotypes of SAM are presented in Table 1.

### 4.2. HCM-Related SAM: Classic Mechanism and Evolving Understanding

HCM-related SAM represents the prototype dynamic obstruction, but current understanding extends substantially beyond simple septal bulging. Cardiovascular magnetic resonance studies in sarcomeric gene mutation carriers have demonstrated dyskinesia of the entire aorto-mitral apparatus, with elongation of the anterior mitral valve leaflet (AMVL) detectable even in subclinical HCM before the development of left ventricular hypertrophy (LVH) [4].

The mechanisms driving SAM in HCM involve complex flow-structure interactions. Computational fluid dynamics studies reveal that late-diastolic vortices generated during mitral inflow pre-position elongated mitral leaflets anteriorly—a phenomenon termed “diastolic anterior motion” (DAM)—creating anatomic substrate for systolic engagement. Drag forces from anteriorly directed flow over the posterior leaflet surface, combined with Venturi lift forces that accelerate as the LVOT narrows, generate the anterior propulsion [8].

#### 4.2.1. Anatomical Variants in HCM-SAM

While anterior mitral leaflet SAM predominates, posterior leaflet SAM represents a rare but distinct anatomical variant. Cases of isolated posterior mitral leaflet elongation causing LVOT obstruction demonstrate that the relationship between leaflet morphology and hemodynamic consequence is highly individualized [9]. Three-dimensional transesophageal echocardiography (3D TEE) has revolutionized visualization of these complex interactions, revealing phenomena such as the “dolphin smile” configuration and double-orifice LVOT created by residual leaflet contact [10].

#### 4.2.2. Dynamic Quantification and Prognostic Assessment

The late-systolic distance between mitral leaflet tip and anterior septum (TISls) measured in apical 3-chamber view has emerged as a powerful quantitative parameter for identifying dynamic LVOTO in HCM [6]. This measurement achieved area under the ROC curve of 0.914 for identification of LVOTO and demonstrated high diagnostic accuracy for dynamic LVOTO with TISls ≤ 14 mm showing 97% sensitivity and TISls ≤ 9 mm showing 95% specificity [11].

#### 4.2.3. Complex HCM Phenotypes

The coexistence of HCM-related dynamic LVOTO with fixed subaortic obstruction from subaortic membrane represents an exceptionally rare but mechanistically instructive combination [12]. Additionally, dual pathology of HCM with coincident severe aortic stenosis creates a “double-threat” scenario with contradictory hemodynamic imperatives—increased afterload from aortic stenosis promotes LVOTO by increasing left ventricular contractility, while reducing preload paradoxically worsens both lesions [13].

### 4.3. Post-Mitral Valve Repair SAM: Iatrogenic Obstruction and Prevention

#### 4.3.1. Mechanisms of Post-Repair SAM

Post-mitral valve repair (MVR) SAM represents a distinct phenotypic category with fundamentally different anatomical substrate than native HCM-SAM. The development of SAM after repair for myxomatous mitral valve disease reflects altered geometric relationships created by annuloplasty ring placement and leaflet modifications. Pre-repair transesophageal echocardiography (TEE) identifies patients at risk through measurement of coaptation point-to-septum distance (C-Sept) and anterior-to-posterior leaflet length ratios. Patients developing post-repair SAM/LVOTO demonstrated smaller anterior/posterior leaflet ratios (0.99 vs. 1.95, *p* < 0.0001) and reduced C-Sept distances (2.53 vs. 3.01 cm, *p* = 0.012) compared to those remaining free of obstruction. These measurements reflect malposition of the coaptation point anteriorly toward the LVOT, creating anatomic substrate for flow-leaflet interaction even in the absence of septal hypertrophy [14].

#### 4.3.2. Risk Stratification and Preventive Interventions

Modern mitral repair techniques incorporate explicit prevention of post-repair SAM through LVOT modification. In a large series of 800 totally endoscopic robotic mitral valve repairs (TERMVR), patients with moderate or high SAM risk (23.8% of total) underwent LVOT modification consisting of ventricular septal bulge myectomy and/or septal myocardial trabeculations resection in 73.2% of high-risk cases. This approach prevented any subsequent need for mitral valve repair revision for SAM, with only one transient SAM episode occurring while on inotropes [15].

#### 4.3.3. Post-Repair SAM and Underlying HCM

A particularly challenging subset involves patients with underlying HCM who develop progressive dynamic LVOTO after mitral valve repair. One case report described a 66-year-old woman with prior mitral repair who developed symptomatic post-repair dynamic LVOTO refractory to medical management, responding dramatically to mavacamten (cardiac myosin inhibitor) with gradient reduction from 33 mmHg at rest to 7 mmHg [16]. However, such isolated cases should serve only an illustrative function and cannot be interpreted as clinical evidence supporting off-label treatment recommendations outside established trial populations.

#### 4.3.4. Post-Repair SAM in Mitral Annular Calcification

Systolic anterior motion can develop in the setting of severe mitral annular calcification (MAC), representing a distinct anatomical substrate. Patients with MAC and SAM demonstrated smaller left ventricular outflow tract diameter, longer anterior mitral leaflet, and greater displacement of the mitral valve coaptation point toward the interventricular septum compared to MAC patients without SAM [17]. Using the ratio of anterior leaflet length to coaptation-to-septum distance effectively separated MAC patients with SAM from those without.

### 4.4. SAM and Dynamic LVOTO After Aortic Valve Replacement (AVR) and TAVR

#### 4.4.1. Post-AVR Dynamic LVOTO: Mechanisms and Hemodynamic Context

Development of dynamic LVOTO and SAM after surgical aortic valve replacement (AVR) represents a paradoxical phenomenon: relief of the fixed outflow obstruction unmasks dynamic obstruction. The integrated mechanism involves the mitral-aortic annular plane angle (normally ≥ 120 degrees), whereby angles < 120 degrees position the mitral orifice more anteriorly, placing mitral leaflets closer to the ventricular septum [18]. A coaptation-to-septum distance < 2.5 cm and ventricular hypertrophy > 15 mm represent unifying anatomical features predisposing to post-AVR LVOTO regardless of underlying diagnosis.

Chronic aortic stenosis maintains elevated intra-ventricular pressure, which prevents septal inward motion and keeps the LVOT unobstructed. After valve replacement, this pressure relief paradoxically allows increased LVOT flow velocity and negative transmitral pressure gradients that draw the anterior leaflet anteriorly [19].

#### 4.4.2. Post-TAVR SAM and Intraoperative SAM Following Surgical AVR Requiring MitraClip: Emerging Recognition of a Rare Complication

Transcatheter aortic valve replacement (TAVR) can precipitate severe dynamic LVOTO with SAM through similar hemodynamic mechanisms as surgical AVR but with potentially different anatomical substrates. Published case reports of post-TAVR SAM illustrate how acute pressure gradient reversal between the left atrium and LVOT can pull the anterior leaflet forward, providing useful pedagogical insights into acute post-procedural management. Similarly, reports describing the use of transcatheter edge-to-edge repair (TEER) with MitraClip for intraoperative SAM following bicuspid aortic valve replacement exemplify rescue options in extreme, refractory scenarios. However, these isolated cases highlight physiological mechanics during emergency stabilization and should not be construed as established clinical standards for routine post-AVR LVOTO management [19,20,21].

### 4.5. Transient SAM in Acute Hemodynamic States

#### 4.5.1. Takotsubo Syndrome with Dynamic LVOTO

Takotsubo syndrome (TTS), also termed stress-induced cardiomyopathy, can present with dynamic LVOTO and SAM-related hemodynamic instability in a subset of patients [22]. The prevalence of cardiogenic shock in TTS ranges from 6 to 20%, and dynamic LVOTO contributes to hemodynamic compromise in approximately 25% of cases [23]. The mechanism differs fundamentally from HCM-SAM: instead of abnormal septal/leaflet anatomy, transient regional wall motion abnormalities (apical akinesia with basal hyperkinesis) create flow acceleration and negative pressure gradients that pull the leaflet anteriorly.

Early recognition of LVOTO in TTS is crucial because management contradicts standard cardiogenic shock protocols: inotrope agents must be avoided as they increase basal hypercontractility and worsen LVOTO, while careful volume expansion and beta-blockade reduce LVOT obstruction by decreasing contractility and increasing diastolic filling time [22,24].

#### 4.5.2. Acute Myocardial Infarction with Transient SAM

Ischemia-induced and reversible SAM without left ventricular hypertrophy represents an exceptionally rare phenomenon. A case of 54-year-old woman with NSTEMI demonstrated transient apical dysfunction and basal hyperkinesis causing dynamic LVOTO despite absent LVH (interventricular septum 8 mm; lateral wall 7 mm, well below diagnostic cutoff), with SAM-related moderate mitral regurgitation and LVOT gradient of 30 mmHg [1]. Complete resolution of SAM, mitral regurgitation, LVOT gradient, and apical wall-motion abnormalities occurred within three weeks, confirming the transient hemodynamic substrate underlying this rare presentation.

#### 4.5.3. Catecholamine-Induced Transient Dynamic LVOTO

Intensive use of inotropic support with norepinephrine and dobutamine can precipitate acute dynamic LVOTO with SAM in acute myocardial infarction. In one case, a 76-year-old patient with acute anterior MI developed profound hemodynamic compromise with new cardiac murmur attributable to SAM and dynamic LVOTO secondary to aggressive inotropic support [25]. Recognition of the underlying mechanism and cessation of inotropes with aggressive fluid resuscitation rapidly stabilized hemodynamics, preventing further deterioration and unnecessary interventions.

### 4.6. Congenital and Anatomic SAM Without Classic HCM

#### 4.6.1. SAM from Elongated Anterior Mitral Leaflet Without Septal Hypertrophy

A distinct phenotype involves SAM and severe LVOTO caused exclusively by elongated anterior mitral leaflet without underlying hypertrophic cardiomyopathy. A young male patient presented with exertional dyspnea (NYHA Class 2–3) and was found to have severe dynamic LVOTO at rest (peak velocity 4.96 m/s, pressure gradient 98.44 mmHg) without HCM. Transthoracic echocardiography showed concentric remodeling of the left ventricle without hypertrophy, while anterior and posterior mitral leaflet lengths were 3.7 cm and 1.3 cm respectively (exceeding normal cutoffs of 3 cm and 1.5 cm) [26]. The patient developed concentric remodeling as a compensatory response to chronically decreased cardiac output from LVOTO.

#### 4.6.2. Congenital Mitral Valve Abnormalities and Accessory Mitral Tissue

Accessory mitral valve tissue (AMVT), an embryological remnant involving the mitral apparatus, can present with severe LVOTO and mitral regurgitation mimicking other cardiac pathologies. These lesions may associate with other congenital anomalies including ventricular septal defect, mitral supravalvular ring, and subaortic membrane. Classification based on anatomical attachment distinguishes supravalvular-level attachments (type-1), leaflet-level attachments either freely swinging or attached to other structures (type-2), and infravalvular-level attachments (type-3) [27]. Functional mitral regurgitation from SAM (driven by Venturi effect of LVOT gradient) appears more likely in type-2A AMVT, while valvular tethering and distortion predominate in type-1 and type-3 lesions.

#### 4.6.3. SAM in Ebstein’s Anomaly

Left ventricular outflow tract obstruction from SAM can uncommonly arise in Ebstein’s anomaly when dilatation of the right atrium causes leftward bulging of the intraventricular septum. A 17-year-old male with Ebstein’s anomaly presented with exertional fatigue and breathlessness, with transthoracic echocardiography revealing significant right atrial dilatation and leftward septal bulging. The anterior mitral valve leaflet was elongated with accessory chordal apparatus demonstrating systolic anterior motion, creating left ventricular outflow tract obstruction through a mechanism similar to HCM [28].

#### 4.6.4. SAM in Congenital d-Transposition of Great Arteries

In d-transposed great arteries post-atrial switch (d-TGA/AtS), sub-pulmonic SAM of the mitral valve can develop through mechanisms distinct from both HCM and acquired obstruction. Among 18 adult d-TGA/AtS patients, those with leaflet SAM (n = 5) compared to those without (n = 13) had significantly higher mitral valve protrusion height (2.1 ± 0.4 vs. 1.5 ± 0.4 cm, *p* ≤ 0.01) and longer anterior mitral valve leaflet length (3.0 ± 0.24 vs. 2.6 ± 0.34 cm, *p* ≤ 0.05) [29]. Higher sub-pulmonic left ventricular ejection fraction in the SAM group (68 ± 5 vs. 54 ± 7%, *p* ≤ 0.005) contributed to SAM development, while systemic right ventricular size, chest deformity, and septal thickness did not correlate with SAM occurrence.

### 4.7. Midventricular Obstruction Without True Leaflet–Septal Contact

#### 4.7.1. Mechanisms Distinguishing Midventricular from Subaortic Obstruction

Midventricular obstruction (MVO) represents a distinct form of LVOTO in HCM, occurring without necessarily involving direct mitral-septal contact. Among 108 patients with apical aneurysms, 103 (95%) had mid-LV obstruction with complete mid-LV systolic emptying, while only 19% demonstrated Doppler systolic gradients ≥ 30 mmHg despite 84% showing midsystolic Doppler signal void (complete flow cessation marker) [30]. This observation indicates that obstruction can occur through papillary muscle-mediated mechanisms distinct from classic SAM-related LVOTO.

#### 4.7.2. Geometric Substrate of Midventricular Obstruction

Compared to control groups, patients with midventricular obstruction had smaller short-axis systolic areas (*p* < 0.007), greater percent short-axis area change (*p* < 0.005), larger papillary muscle areas (*p* < 0.003), and greater diastolic papillary muscle areas relative to short-axis diastolic areas (*p* < 0.005) [30]. Complete emptying occurs circumferentially around central papillary muscles that contribute to obstruction—a mechanism fundamentally different from anterior displacement of mitral leaflets by flow and septal drag.

#### 4.7.3. Apical Aneurysm Formation and Midventricular Obstruction

Computational finite-element modeling of left ventricular stress patterns revealed that myofiber stress in midventricular obstruction models was 75.0% higher than in subaortic obstruction models (654.5 kPa vs. 373.9 kPa). The maximum stress on the left ventricular apex increased 79.9%, 69.3%, and 117.8% compared to the surrounding myocardium in healthy, subaortic obstruction, and midventricular obstruction models, respectively [31]. These biomechanical findings suggest that midventricular obstruction may directly initiate apical aneurysm formation through locally elevated myofiber stress at the apex.

## 5. Diagnostic Approaches

### 5.1. Echocardiographic Assessment and Two-Dimensional Imaging

Echocardiography remains the primary diagnostic modality for symptomatic patients suspected of presenting with SAM. Innovations in two-dimensional transthoracic echocardiography (2D-TTE) and Doppler technology have markedly enhanced the quantification of dynamic LVOTO. Standard 2D imaging typically delineates the characteristic mid-systolic anterior excursion of the mitral valve toward the septum. The severity of the dynamic gradient can be reliably measured using continuous-wave Doppler, optimized through strategic, multiple-window acoustic views. Characteristically, dynamic LVOTO displays a late-peaking, “dagger-shaped” continuous-wave Doppler profile, which serves as a pathognomonic diagnostic feature [32].

### 5.2. Three-Dimensional Echocardiography and Advanced Imaging

Three-dimensional transesophageal echocardiography (3D-TEE) has substantially improved the spatial characterization of severe mitral regurgitation secondary to SAM. This modality provides highly accurate anatomical rendering and dynamic modeling of the valvular structures. Precise volumetric rendering allows for detailed mapping of leaflet heights, coaptation lines, and papillary muscle spatial orientation relative to LVOT geometry. Furthermore, 3D imaging can visualize transient structural interactions, such as the “dolphin smile” configuration and the formation of a dual-orifice LVOT caused by redundant, billowing leaflet tissue during systole [10,33].

Three-dimensional epicardial echocardiography provides valuable utility when TEE is contraindicated or when acoustic windows are suboptimal. In patients with HOCM undergoing surgical septal reduction, 3D epicardial imaging demonstrates high correlation with TEE measurements regarding structural dimensions [34]. Concordance is particularly robust for quantifying septal wall thickness, calculating the area of the LVOT, and pinpointing the exact pivot point of SAM. Consequently, intraoperative TEE and epicardial imaging serve as essential roadmaps for initial anatomical assessment, real-time procedural monitoring, and post-myectomy quality control [35].

### 5.3. Specialized Anatomical Assessment

Anatomical variants identified via 3D-TEE elucidate the specific susceptibility to SAM and MR in HOCM populations. Cleft-like indentations (CLIs) of the mitral valve have been observed in up to 93.3% of HOCM patients compared to 39% of control subjects. Crucially, deep CLIs are present in 85.6% of the HOCM phenotype versus only 25.4% in controls [36]. The presence of these structural variants does not correlate with the severity or direction of pre-operative MR, nor do they typically mandate surgical correction during myectomy. However, true structural clefts—as opposed to simple indentations—are significantly associated with anteriorly directed MR jets, necessitating precise diagnostic differentiation [37].

### 5.4. Provocative Testing and Exercise-Induced LVOTO: Diagnostic and Prognostic Significance

#### 5.4.1. Postprandial Echocardiography as a Powerful Provocative Maneuver

The identification of latent LVOTO in HCM patients requires comprehensive provocative testing strategies beyond conventional resting measurements. Postprandial echocardiography has emerged as a remarkably effective technique for unmasking hemodynamically significant gradients in patients who appear non-obstructive at baseline. Among 252 consecutive HCM patients without resting LVOTO, postprandial LVOT gradients were substantially higher compared with routine echocardiography at rest (median 9.0 versus 0 mmHg, *p* < 0.0001) and with Valsalva maneuver provocation (18.5 versus 1.5 mmHg, *p* < 0.0001). The clinical significance of this finding was substantial: while 49 patients (19.5%) achieved the ≥50 mmHg threshold under routine conditions, an additional 90 patients (35.7%) manifested postprandial gradients ≥ 50 mmHg, with 38 (15.1%) demonstrating significant obstruction exclusively during postprandial stress echocardiography. Notably, 71 of these patients were treated with invasive or enhanced drug therapies including surgical myectomy, alcohol septal ablation (ASA), disopyramide, and mavacamten, with 32 patients (45.1%) having gradients ≥ 50 mmHg only after eating and 8 (11.3%) exclusively with postprandial stress exercise [38]. This paradigm-shifting evidence demonstrates that eating constitutes a powerful physiological provocative tool that should be systematically integrated into HCM evaluation protocols, as it identifies a substantial proportion of patients who would otherwise be misclassified as having non-obstructive disease.

#### 5.4.2. Combined Cardiopulmonary Exercise Testing and Stress Echocardiography: Mechanistic Insights

Integrating cardiopulmonary exercise testing (CPET) with simultaneous stress echocardiography (CPETecho) enables comprehensive assessment of both dynamic hemodynamics and functional capacity, revealing distinct exercise intolerance mechanisms across HCM phenotypes. In a multicenter cohort of 195 HCM patients undergoing maximal semi-supine CPETecho, the diagnostic yield for dynamic LVOTO detection was substantial, with highest gradients occurring post-exercise recovery rather than during peak exertion (*p* < 0.001). Exercise intolerance, defined as peak VO_2_ < 80% predicted, affected 52% overall, but with markedly different prevalence between obstructive (65%) and non-obstructive (35%) phenotypes (*p* < 0.001). Critical mechanistic insights emerged: in obstructive HCM, LVOTO gradient was the primary determinant of exercise intolerance, while in non-obstructive disease, mid-exercise E/e’ ratio (diastolic dysfunction marker) correlated most strongly with functional limitation. Chronotropic incompetence was prevalent in 61% of patients, frequently attributable to beta-blocker use (46%) [39]. These findings underscore the fundamental principle that exercise physiology in HCM is heterogeneous and phenotype-dependent, requiring integrated assessment strategies rather than singular measurements.

#### 5.4.3. Determinants of Functional Capacity: Exercise-Induced Diastolic Dysfunction and Pulmonary Pressures

Contemporary research has illuminated the complex hemodynamic determinants of exercise tolerance in HCM through multiparameter assessment during stress testing. Among 388 HCM patients (46% obstructive) undergoing baseline CPET-stress echocardiography with median 7.4-year follow-up, 16.2% experienced heart failure hospitalization or progression to end-stage HCM. Patients who progressed had greater left atrial dimensions at rest (45.0 versus 41.0 mm, *p* < 0.001) and indexed volumes (36.4 versus 29.0 mL/m^2^, *p* < 0.001) with further dilation during stress (*p* = 0.046). Critically, during exercise stress testing, these patients exhibited lower peak oxygen consumption (16.0 versus 19.5 mL/kg/min, *p* = 0.001), reduced percent-predicted VO_2_ (*p* = 0.006), earlier anaerobic threshold (*p* = 0.032), impaired ventilatory efficiency (*p* = 0.048), and greater chronotropic incompetence (*p* < 0.001). Multivariate analysis identified dyslipidemia, higher E/e’ ratio, and lower peak VO_2_ as independently associated with the primary outcome, with reduced peak VO_2_ demonstrating the strongest association [40]. The identification of exercise-induced diastolic dysfunction and elevated pulmonary pressures as independent predictors of clinical deterioration has profound implications for risk stratification and therapeutic decision-making in HCM.

#### 5.4.4. Stress Echocardiography: Beyond LVOTO Detection

Stress echocardiography in HCM has historically focused narrowly on LVOTO quantification; however, contemporary evidence demonstrates substantially greater diagnostic and prognostic value through comprehensive phenotyping. The Stress Echo 2020 study prospectively enrolled 235 HCM patients (mean age 48 ± 15 years, 113 men) for comprehensive stress echocardiography assessment incorporating seven sequential evaluation steps: detection of new regional wall motion abnormalities (Step A), coronary flow velocity reserve (CFVR) assessment via Doppler echocardiography (Step D), heart rate reserve quantification (Step E), mitral regurgitation flow characterization (Step F), and LVOTO gradient measurement (Step G). Technical success rates were uniformly high except for CFVR assessment (80% with semi-supine bicycle, 100% with adenosine post-treadmill) and mitral regurgitation quantification (99%). The prevalence of abnormalities ranged from 73% for chronotropic incompetence (heart rate reserve < 1.80) to only 1% for inducible regional wall motion abnormalities, with intermediate rates for impaired CFVR < 2.0 (44%), moderate-to-severe mitral regurgitation (32%), and significant LVOTO (>50 mmHg, 26%) [41]. Generation of a comprehensive stress echo score from 0 (all parameters normal) to 5 (all abnormal) revealed that 86% of patients demonstrated abnormal findings on one or two parameters, while only 8% displayed three or more abnormalities. This multidimensional approach permits true phenotypic characterization of the heterogeneous manifestations of HCM, facilitating personalized functional assessment and enabling identification of clinically silent but hemodynamically important abnormalities.

#### 5.4.5. Stress-Induced Pulmonary Congestion: A Novel Risk Marker

An emerging and underappreciated phenomenon is exercise-induced pulmonary congestion detectable by lung ultrasound during stress echocardiography, reflecting inadequate preload reserve and diastolic decompensation [42]. Among 128 HCM patients undergoing exercise stress echocardiography with concurrent lung ultrasound assessment in 10 quality-controlled centers, B-lines indicating pulmonary congestion were present in 13 patients at rest (10%) but in 38 during stress (30%, *p* < 0.0001). Patients with stress-induced B-lines exhibited higher resting E/e’ ratios (14 ± 6 versus 11 ± 4, *p* = 0.016) and systolic pulmonary artery pressure (33 ± 10 versus 27 ± 7 mmHg, *p* = 0.002). At peak exercise, these patients demonstrated higher peak E/e’ ratios (17 ± 6 versus 13 ± 5, *p* = 0.003), markedly elevated stress systolic pulmonary artery pressure (55 ± 18 versus 40 ± 12 mmHg, *p* < 0.0001), reduced preload reserve (68 versus 30%, *p* = 0.001), and greater increase in mitral regurgitation (42 versus 17%, *p* = 0.013). Among baseline parameters, B-line count and systolic pulmonary artery pressure were the only independent predictors of exercise-induced pulmonary congestion. Importantly, two-thirds of HCM patients developing pulmonary congestion during exercise demonstrated no evidence of B-lines at rest, indicating that functional stress assessment uniquely unmasks subclinical diastolic impairment with substantial prognostic implications [42].

#### 5.4.6. Left Atrial Strain as a Predictor of Exercise Capacity and Clinical Progression

Left atrial function, assessed through strain imaging during stress echocardiography, represents a novel marker of exercise tolerance and risk of clinical deterioration requiring interventional therapy [43,44]. In a prospective cohort of 536 HCM patients undergoing concurrent exercise stress echocardiography and cardiopulmonary exercise testing, left atrial contractile strain emerged as an independent predictor of exercise intolerance (defined as lowest quartile of percent-predicted peak VO_2_, ≤52%) with optimal receiver operating characteristics analysis threshold of −13.8% (area under curve 0.68, *p* < 0.001). Multivariable logistic regression confirmed impaired left atrial contractile strain as independently predictive of exercise intolerance (*p* < 0.001). More clinically impactful, among 258 patients followed longitudinally, those with left atrial stiffness exceeding the median value (E/e’ ratio/left atrial reservoir strain ratio ≥ 0.41) demonstrated significantly higher probability of requiring septal myectomy compared to those below the median (*p* = 0.02) [44]. These findings indicate that left atrial phenotyping through strain imaging during stress testing provides mechanistic insight into functional limitation and offers predictive capability for identifying patients destined to require definitive interventional therapy.

### 5.5. Cardiac Magnetic Resonance Imaging: Fibrosis Assessment and Risk Stratification

#### 5.5.1. Late Gadolinium Enhancement: The Gold Standard for Myocardial Fibrosis Quantification

Cardiac magnetic resonance imaging with late gadolinium enhancement (LGE) has emerged as the gold standard for non-invasive myocardial fibrosis quantification in HCM [45], providing critical prognostic information that directly influences risk stratification and therapeutic decision-making. Among 436 prospectively enrolled HCM patients (median age 58 years, 72.2% men) followed for median 11 years, late gadolinium enhancement was present in 87% (381 patients), with 21% meeting the endpoint of all-cause mortality. Multivariate Cox regression analysis demonstrated that absolute LGE extent (as percentage of left ventricular mass) was an independent predictor of all-cause mortality (*p* < 0.001). For each 1% increment in LGE extent, mortality risk relative to a patient without LGE increased by 5.9% (95% CI: 2.9–9%). HCM patients with 10% and 20% LGE burden demonstrated respective 1.8-fold and 3.1-fold increases in mortality risk compared to those without LGE on CMR imaging. Novel threshold values stratifying risk were derived: low-risk (≤3.27%), medium-risk (>3.27% and <11.08%), and high-risk (≥11.08%) groups based on LGE burden, with stratification yielding a 5-fold increase in mortality risk comparing high-risk and low-risk LGE groups (hazard ratio 5.87, 95% CI: 2.04–16.95, *p* = 0.001) [46]. These findings underscore the central importance of comprehensive LGE quantification in HCM risk stratification, with particular relevance for identifying high-risk patients who may benefit from earlier invasive intervention or device therapy.

#### 5.5.2. Advanced CMR Tissue Characterization: T1 Mapping, Extracellular Volume, and Strain Analysis

Beyond conventional LGE assessment, advanced CMR techniques including native T1 mapping, extracellular volume fraction (ECV) measurement, and myocardial strain analysis provide incremental prognostic information. In a prospective case–control study evaluating 150 HCM patients and 100 age- and sex-matched healthy controls, HCM patients demonstrated significantly impaired strain and torsion metrics compared with controls: global longitudinal strain (−16% versus −20%), circumferential strain (−18% versus −21%), radial strain (29% versus 38%), and global left ventricular torsion (1.27°/cm versus 1.95°/cm), all *p* < 0.001. These abnormalities were observed even in late gadolinium enhancement-negative patients, suggesting early functional remodeling preceding overt fibrosis. Global left ventricular torsion demonstrated the highest diagnostic performance for LGE detection (area under curve 0.995), surpassing global longitudinal strain (0.877), native T1 (0.731), and extracellular volume (0.657). A cut-off value of 0.7°/cm provided optimal sensitivity and specificity and was associated with adverse prognosis in survival analysis [47]. These findings demonstrate that CMR-derived strain and torsion parameters detect early myocardial dysfunction in HCM beyond conventional markers, with global left ventricular torsion emerging as a sensitive and robust non-invasive marker with diagnostic and prognostic potential.

#### 5.5.3. Serial CMR Assessment: Tracking Fibrosis Progression and Functional Decline

Sequential CMR imaging in HCM patients reveals dynamic changes in myocardial architecture that correlate with clinical progression and predict adverse outcomes. Among 114 HCM patients undergoing two CMR scans over a median 5.1-year interval (interquartile range 3.5–6.5), late gadolinium enhancement extent increased from 0.5% of left ventricular mass (interquartile range 0.1–3.6%) to 1.6% (0.5–6.1%, *p* < 0.001). Changes in longitudinal, circumferential, and radial strain values were significant from first to second CMR scan, with several correlations emerging between absolute changes in strain values and increases in LGE mass, including longitudinal strain (β = 0.227, *p* = 0.016) and circumferential strain (β = 0.421, *p* < 0.001). During 4.3-year median follow-up after the second CMR (interquartile range 2.2–6.9), 40 patients experienced a composite endpoint including sudden cardiac death, life-threatening ventricular arrhythmias, stroke, new-onset atrial fibrillation, and heart failure hospitalizations. Among patients with LGE < 15% at baseline, yearly absolute changes in radial short-axis strain predicted outcomes beyond baseline HCM score and LGE extent (hazard ratio 1.12 per 1% change, 95% confidence interval 1.03–1.22, *p* = 0.011) [48]. These findings establish serial CMR-based strain analysis as a valuable tool for detecting subtle variations in left ventricular systolic function associated with fibrosis progression and adverse clinical trajectory.

#### 5.5.4. CMR in Phenotype Differentiation and Phenocopy Recognition

The critical role of CMR extends beyond HCM diagnosis to accurate distinction from phenocopies—conditions presenting with HCM-like hypertrophic phenotypes but fundamentally different etiologies requiring distinct therapeutic approaches. CMR accurately quantifies hypertrophy extent and distribution, characterizes myocardial tissue through late gadolinium enhancement patterns and T1 mapping, and detects associated abnormalities. In the context of phenocopies, CMR facilitates differentiation between sarcomeric HCM and other diseases including cardiac amyloidosis (characterized by distinct diffuse and symmetric subendocardial late gadolinium enhancement with apical sparing on longitudinal strain), Anderson–Fabry disease (with low T1 values), and mitochondrial cardiomyopathies. The non-invasive diagnostic capability of CMR provides essential guidance for clinical decision-making and management strategy selection, highlighting the imperative for comprehensive multimodal imaging in all patients presenting with unexplained left ventricular hypertrophy [49].

## 6. Management of Obstructive Hypertrophic Cardiomyopathy

### 6.1. Evolution and Scope of Current Guidelines

The 2024 AHA/ACC/AMSSM/HRS/PACES/SCMR Guideline for the Management of Hypertrophic Cardiomyopathy represents the most recent comprehensive update of evidence-based recommendations for clinicians managing patients with this condition [50]. These guidelines underwent a comprehensive literature search from September 2022 through November 2022, encompassing studies, reviews, and clinical evidence from PubMed, EMBASE, the Cochrane Library, and other authoritative databases. Additional relevant studies published through May 2023 during the guideline writing process were incorporated into the evidence tables. This extensive review process resulted in updated recommendations that build upon the foundational 2020 AHA/ACC Guideline for the Diagnosis and Treatment of Patients with Hypertrophic Cardiomyopathy, incorporating significant new evidence that has emerged over the intervening years.

The scope of contemporary guidelines extends beyond septal reduction therapies to encompass risk stratification for sudden cardiac death, management of atrial fibrillation, reproductive counseling for genetically affected individuals, and emerging therapeutic innovations. The guidelines emphasize a personalized approach based on genotype and phenotype, recognizing that hypertrophic cardiomyopathy encompasses diverse molecular subtypes with varying disease trajectories and treatment responses [51]. This shift toward precision medicine reflects the expanding understanding of HCM pathobiology and improving capability to identify disease-modifying strategies.

### 6.2. Treatment Hierarchy and Septal Reduction Recommendations

Septal myectomy (SM) occupies a central position in the AHA/ACC therapeutic hierarchy for obstructive HOCM, receiving a Class I recommendation with Level of Evidence B-NR (Non-randomized) [52]. This evidence rating reflects the strength of outcome data supported by numerous observational studies and extensive clinical experience from specialized centers, despite the lack of randomized controlled trials. The non-randomized evidence base, while robust, acknowledges the practical and ethical constraints to randomizing symptomatic patients requiring intervention to different treatment modalities.

Surgical myectomy is considered the gold standard treatment for symptomatic patients with hemodynamically significant obstruction refractory to maximal medical therapy. The procedure receives strong guideline support based on its proven ability to eliminate left ventricular outflow tract (LVOT) gradients, resolve systolic anterior motion (SAM) of the mitral valve, and improve functional symptoms across multiple observational cohorts. The technical refinement of extended myectomy, originally described by Messmer in 1994, allows comprehensive addressing of septal hypertrophy and associated mitral valve abnormalities through a single surgical intervention [52].

### 6.3. Nuanced Selection Between Septal Myectomy and Alcohol Septal Ablation

Rather than a simple dichotomy between surgical and percutaneous approaches, contemporary management requires a nuanced, patient-tailored selection process based on anatomical, clinical, and procedural factors.

Surgical Septal Myectomy (SM) remains the intervention of choice when complex left ventricular outflow tract (LVOT) anatomy is present. This includes severe asymmetric septal hypertrophy (>30 mm), intrinsic mitral valve abnormalities (e.g., marked leaflet elongation or anterior displacement), and abnormal papillary muscle insertion, which cannot be addressed percutaneously. Furthermore, SM offers lower rates of permanent pacemaker implantation and a slightly higher likelihood of complete gradient elimination in young, low-risk patients.

Alcohol Septal Ablation (ASA) is a well-established alternative that yields long-term survival and symptomatic relief comparable to surgery in appropriately selected candidates. Ideal candidates are older adults or patients with significant comorbidities, provided they possess suitable septal coronary anatomy (i.e., an accessible major septal perforator branch supplying the site of SAM-septal contact) and isolated basal septal hypertrophy without structural mitral valve pathology. Both European and North American guidelines increasingly emphasize that procedural outcomes for both ASA and SM are heavily volume-dependent, recommending that both therapies be restricted to comprehensive HCM centers of excellence to optimize success rates and minimize periprocedural complications [50,52,53].

## 7. Surgical Management in Hypertrophic Cases

### 7.1. Septal Myectomy

Extended septal myectomy is the established gold-standard surgical intervention for HOCM patients presenting with hemodynamically significant LVOTO [54]. The procedure can be performed via a transaortic or transseptal approach. The primary objective of an extended myectomy is to surgically debulk the hypertrophied basal septum, thereby widening the outflow tract and eliminating the high-velocity drag forces (Venturi effect) that drive the anterior leaflet into the LVOT. A seminal series utilizing the Messmer concept highlighted the durability of this approach: among 84 patients undergoing extended myectomy, only one patient required concomitant mitral valve surgery for intrinsic degenerative disease. At hospital discharge, residual MR greater than mild was documented in only 5% of the cohort, and long-term follow-up demonstrated exceptional survival free from cardiac re-operation [55].

A critical intraoperative decision involves calibrating the depth and extent of myocardial resection to ensure optimal gradient relief while avoiding complications such as ventricular septal defects. The contemporary extended myectomy involves excavating a rectangular trough of septal muscle, extending from just below the aortic valve commissures down to the base of the papillary muscles. The width of the resection is tailored to pre-operative imaging and intraoperative TEE findings. Modern iterations of this technique have consistently achieved low operative mortality and substantial reductions in resting and provoked gradients. Real-time intraoperative TEE allows the surgical team to evaluate the residual gradient immediately post-cardiopulmonary bypass, ensuring a low resting gradient and the complete eradication of provocable obstruction [55].

Regarding long-term gradient reduction, surgical myectomy demonstrates clear superiority over percutaneous alternatives. Compared to alcohol septal ablation, myectomy achieves a significantly greater reduction in peak instantaneous gradients both at rest and during the Valsalva maneuver, higher rates of MR resolution, and superior functional improvement in NYHA class (*p* < 0.001). Hemodynamic improvements following extended myectomy are highly durable, with mean gradient reductions maintained at 49.3 ± 17.0 mmHg at mid-term follow-up and sustained beyond 10–12 years [56].

### 7.2. Anatomical Variations and Additional Procedures Beyond Myectomy

For the majority of HOCM patients with symptomatic LVOTO, isolated septal myectomy provides definitive therapeutic relief. However, on a selective basis, specific anatomical variations dictate concomitant interventions on the subvalvular apparatus. In patients presenting with co-existing intrinsic, degenerative mitral valve disease (e.g., chordal rupture or leaflet prolapse), simultaneous mitral valve repair or replacement is indicated. A diverse surgical armamentarium has been developed to address complex anatomy, including anterior leaflet plication, leaflet elongation patches, posterior leaflet height reduction, and papillary muscle repositioning or reorientation [2,57].

The decision to perform auxiliary mitral interventions requires meticulous intraoperative assessment. Mitral valve replacement should be strictly avoided unless independent, irremediable degenerative pathology is present. Geometric indicators for concomitant repair include an excessively elongated or redundant posterior leaflet that displaces the coaptation point anteriorly, anomalous chordal insertions directly into the anterior leaflet, or persistent SAM post-initial septal resection. These tailored techniques preserve the native valve architecture and optimize long-term left ventricular performance [58].

In cases characterized by elongated or displaced papillary muscles, repositioning the papillary muscle heads can prevent obstructive mid-systolic folding into the LVOT. This surgical approach involves mobilizing and anchoring the papillary muscle heads posteriorly toward the left ventricular free wall, away from the septum. For patients with severe pre-operative MR and restricted leaflet mobility, targeted chordal cutting can effectively reduce the tenting area and mitigate regurgitation. Comprehensive planning requires assessing septal width, papillary muscle geometry, leaflet height, and the systolic coaptation point [59].

### 7.3. Edge-to-Edge Mitral Valve Repair Combined with Myectomy

The surgical Alfieri edge-to-edge technique is an anatomy-dependent adjunct rather than a routine treatment, utilized selectively during septal myectomy. This technique involves placing a structural suture between the mid-adversaria of the anterior and posterior mitral leaflets, creating a double-orifice mitral valve configuration. This modification mechanically restricts the systolic excursion (or “billowing”) of the anterior leaflet into the outflow tract. A comparative study evaluating 35 patients undergoing combined myectomy and edge-to-edge repair (MER) versus 55 patients undergoing isolated myectomy (IMO) demonstrated that at a mean follow-up of 26 months, 87% of patients in both cohorts achieved NYHA class I–II. Notably, the MER group demonstrated a significantly lower incidence of residual or recurrent SAM both early post-operatively and at late follow-up [60].

Mechanistically, the edge-to-edge repair stabilizes the anterolateral leaflet tip, dampening hydrodynamic drag forces. While this technique induces a mild increase in transmitral diastolic gradients, the elevation is clinically negligible (<5 mmHg) and non-progressive, provided the baseline valve area is sufficient. A pilot randomized controlled trial involving 30 HOCM patients confirmed that combined MER and myectomy achieved superior intraoperative stabilization of the valve compared to myectomy alone, without increasing in-hospital or 12-month mortality. Extended septal myectomy alone is the standard and sufficient treatment for most patients presenting with SAM-driven functional mitral regurgitation. Eliminating leaflet–septal contact via robust myectomy addresses the underlying hydrodynamic drag forces and typically restores native valve coaptation without requiring direct mitral manipulation. Concomitant mitral procedures (such as edge-to-edge repair) or replacement must not be performed routinely. Unnecessary mitral intervention carries inherent risks, including increased transmitral diastolic gradients, functional mitral stenosis, or unwarranted prosthetic valve replacement. Therefore, concomitant edge-to-edge repair remains a secondary, highly selective adjunct reserved exclusively for patients with intrinsic structural leaflet pathology, marked redundant leaflet length, or persistent SAM following initial adequate septal resection [61].

### 7.4. Minimally Invasive and Robotic-Assisted Approaches

Robotic-assisted trans-mitral septal myectomy has emerged as a safe and effective alternative to conventional median sternotomy in select patient populations. The trans-mitral vector provides excellent visualization of the subvalvular apparatus and proximal septum through the mitral annulus, facilitating simultaneous papillary muscle reorientation or complex leaflet repair. This procedure is typically performed via a right anterior mini-thoracotomy utilizing a robotic console. This approach is particularly advantageous for patients presenting with moderate septal hypertrophy combined with highly mobile papillary muscles or excessive leaflet height, where the dynamic obstruction is primarily driven by valvular rather than septal components.

Minimally invasive approaches offer significant patient benefits, including attenuated surgical trauma, reduced intraoperative blood loss, minimized cosmetic impact, and accelerated functional recovery. However, these procedures require steep surgical expertise and specialized instrumentation. Rigid patient selection is mandatory; patients presenting with multi-vessel coronary artery disease requiring concomitant coronary artery bypass grafting (CABG), severe aortic valve calcification, or advanced pulmonary disease are not candidates for minimal-access myectomy. In a comparative series of HOCM patients, 17 underwent a totally minimally invasive procedure while 28 underwent standard sternotomy, with both approaches yielding excellent, comparable technical outcomes [62].

In emergent scenarios, minimally invasive techniques provide rapid therapeutic options. Emergency trans-mitral septal myectomy has been successfully deployed following catastrophic complications of alcohol septal ablation, or in patients undergoing high-risk aortic valve interventions who develop acute intraoperative LVOTO. Utilizing a transseptal or trans-mitral approach obviates the need for emergency re-sternotomy or extension of aortic cross-clamp times, providing a versatile option for managing acute hemodynamic collapse associated with acute leaflet fluttering, angina, or refractory heart failure [63].

## 8. Surgical Management in Non-Hypertrophic Cases

### 8.1. Post-Operative SAM Following Mitral Valve Repair

Iatrogenic SAM represents a well-characterized post-operative complication following surgical mitral valve repair for degenerative MR, occurring in 5–10% of cases. The primary anatomical subcomponents predisposing to post-repair SAM include excessive height of the residual posterior leaflet and the surgical implantation of an undersized annuloplasty ring. Recognition of these risk factors has led to the widespread adoption of intraoperative preventive strategies [32].

The etiology of post-repair SAM is fundamentally multifactorial. Aggressive annular undersizing restricts the anteroposterior diameter of the valve, shifting the coaptation line anteriorly into the outflow tract. If a redundant posterior leaflet is left un-resected or un-plicated, it mechanically pushes the anterior leaflet forward during systole. Concurrently, an excessive anterior leaflet length increases the volume of tissue redundant within the LVOT. The clinical spectrum ranges from mild, asymptomatic gradient elevations to severe obstruction (>100 mmHg) precipitating acute low-output syndrome [64].

The identification of acute, severe post-repair MR or LVOTO relies heavily on intraoperative TEE performed by the anesthesia and surgical teams immediately following weaning from cardiopulmonary bypass. Fortunately, a significant proportion of intraoperative SAM cases can be managed conservatively without requiring an immediate return to cardiopulmonary bypass [32].

In the context of congenital heart surgery, acute intraoperative SAM can be unmasked by shunt closures or valvular reconstructions. Conservative intraoperative stabilization algorithms include aggressive volume loading to expand the left ventricle, cessation of inotropic support to minimize hypercontractility, and the administration of short-acting beta-blockers to slow the heart rate and widen the LVOT. Prospective data have demonstrated that the majority of pediatric patients manifesting acute SAM during complex repairs (such as for congenitally corrected transposition of the great arteries) can be successfully managed with this conservative medical strategy, with clinical stabilization typically achieved within 90 min. Severe, refractory SAM is clinically defined by a persistent systolic gradient discrepancy (>30 mmHg) or acute hemodynamic instability [32].

When conservative measures fail, various surgical re-repair techniques are preferred over valve replacement. Options include placing a rescue Alfieri stitch, upsizing the annuloplasty ring to restore proper geometry, or performing posterior leaflet plication. For patients experiencing severe SAM driven by ring undersizing, transitioning to a larger ring or a flexible band can quickly restore the proper coaptation line [64,65].

Late-onset SAM occurring years after an initially successful mitral valve repair is an exceedingly rare but severe clinical entity. This late degradation can be driven by progressive left ventricular reverse remodeling, leaflet elongation, or the development of a sigmoid septum. In documented cases, totally endoscopic ring removal combined with an Alfieri stitch effectively eliminated late-onset SAM and recurrent MR. This underscores the necessity of lifelong echocardiographic surveillance for patients who have undergone surgical mitral valve reconstruction [66].

Preventing post-repair SAM remains a paramount surgical priority. Pre-operative planning must rigorously evaluate the height of the posterior leaflet and the baseline dimensions of the LVOT. Excessive undersizing must be strictly avoided in patients with a narrow or angled outflow tract. Comprehensive TEE assessment before and after the repair is mandatory to evaluate the surgical geometry and ensure optimal outcomes [66].

### 8.2. SAM Associated with Congenital Cardiac Lesions

In pediatric patients undergoing surgical correction for congenital heart defects, such as a partial atrioventricular septal defect (pAVSD), late-onset SAM can develop in the remote post-operative period. Notably, severe LVOTO secondary to SAM can manifest decades after the initial surgery in the complete absence of myocardial hypertrophy, driven primarily by anomalous chordal attachments. Congenital SAM substrates are notoriously refractory to conventional medical therapy. However, open-heart surgical resection of the restrictive, anomalous chordae tendineae represents a definitive treatment, achieving complete resolution of the outflow gradient and long-term symptom relief [6].

Managing late-onset SAM in patients with complex congenitally malformed hearts requires a precise understanding of the altered anatomy. Advanced three-dimensional echocardiography and cardiac magnetic resonance (CMR) imaging are crucial for identifying anomalous chordal insertions and mapping their spatial relations. Furthermore, a thorough review of prior surgical patches, conduits, or synthetic biomaterials is mandatory before planning a re-operation. A transseptal surgical approach frequently offers optimal visualization of the subvalvular structures in these challenging cases [67].

Post-operative SAM can also complicate the repair of a double outlet right ventricle (DORV). Even in patients without pre-existing LVOTO, postoperative factors such as tachycardia, localized septal edema, and aggressive inotropic support can precipitate acute dynamic SAM. Initial management focuses on optimization of fluid status and reduction in inotropes under close TEE surveillance. A return to cardiopulmonary bypass for definitive surgical adjustment is indicated only if profound hemodynamic instability persists or if the patient cannot be safely weaned from mechanical support [67].

### 8.3. SAM Following Aortic Valve Interventions

SAM represents a major clinical challenge when encountered in patients with severe aortic stenosis (AS) undergoing aortic valve interventions, particularly those presenting with marked left ventricular hypertrophy (LVH). Following successful relief of the fixed stenotic obstruction, patients can manifest severe, dynamic LVOTO driven by unmasked SAM, mimicking an obstructive cardiomyopathy phenotype. This “suicide left ventricle” phenomenon occurs because the acute removal of a high-resistance afterload unmasks severe, latent mid-cavity and outflow tract hypercontractility. The hypermobile mitral apparatus is drawn into an outflow tract already narrowed by concentric hypertrophy, precipitating acute cardiogenic shock, severe MR, and life-threatening ventricular arrhythmias requiring emergent mechanical or electrical support [5].

Pre-operative identification of patients at risk for developing a “suicide left ventricle” is critical. High-risk indicators include severe asymmetric septal hypertrophy, marked pre-operative hyperkinesis, or a documented family history of HCM. In high-risk phenotypes, some centers recommend performing a prophylactic septal myectomy at the time of surgical AVR to definitively preempt this complication. Clinical management requires intensive intraoperative hemodynamic monitoring and immediate TEE assessment upon cross-clamp removal [5].

The management of an acute suicide left ventricle may require urgent surgical intervention. The utilization of sutureless aortic valve prostheses (e.g., the Perceval valve) facilitates rapid valve deployment, allowing the surgeon to perform an efficient septal myectomy through the aortic root without excessively prolonging cross-clamp times. Depending on the specific anatomical driver, alternative strategies such as trans-mitral repair or an Alfieri stitch may be deployed. In critically ill patients presenting with profound shock, veno-arterial extracorporeal membrane oxygenation (VA-ECMO) serves as an invaluable bridge to recovery, allowing time for myocardial stunning to resolve and hemodynamics to stabilize prior to definitive repair [68].

## 9. Non-Surgical and Percutaneous Therapeutic Options

### 9.1. Alcohol Septal Ablation (ASA)

Percutaneous alcohol septal ablation (ASA) has established itself as a robust therapeutic alternative to surgical myectomy for HOCM patients with severe symptomatic LVOTO who are refractory to maximally tolerated medical therapy. The procedure entails the targeted delivery of absolute ethanol into a selectively catheterized major basal septal perforator artery. This induces a localized, controlled myocardial infarction, resulting in acute septal akinesia followed by long-term fibrotic thinning and subsequent widening of the LVOT. Contemporary clinical guidelines assign ASA a Class I recommendation as an alternative to surgery in symptomatic individuals who lack secondary structural mitral valve disease requiring operative repair [69].

Large-scale European registry data encompassing 1206 HOCM patients undergoing ASA documented a complete clinical and hemodynamic response (CCHR) in 59% of the cohort at a median follow-up of 4.9 years, with institutional volume serving as a powerful predictor of success [70]. Similarly, data from the Brazilian multicenter BRASA registry highlighted that 92.8% of patients achieved functional optimization to NYHA class I/II. In this cohort, mean resting LVOT gradients decreased profoundly from 88.4 ± 37.2 mmHg to 27.0 ± 21.9 mmHg, accompanied by a reduction in mean interventricular septal thickness from 19.3 ± 4.1 mm to 14.7 ± 3.7 mm [71].

While surgical myectomy typically achieves more complete gradient eradication and higher rates of NYHA class I achievement, ASA offers a less invasive option with reduced initial procedural trauma. However, a comprehensive meta-analysis of 20 studies (4547 patients) demonstrated that while long-term cardiovascular and all-cause mortality were statistically equivalent between myectomy and ASA, periprocedural complications varied significantly. ASA was associated with a lower rate of acute systemic complications but a significantly higher incidence of permanent pacemaker dependency (12.4% vs. 4.31%, *p* = 0.0004) and a greater requirement for future re-intervention (10.1% vs. 0.27%, *p* < 0.001) due to incomplete septal remodeling [72].

### 9.2. Transcatheter Edge-to-Edge Repair (TEER)

Transcatheter edge-to-edge repair (TEER) utilizing clip-based systems (e.g., MitraClip) represents a selective, non-routine percutaneous strategy for treating HOCM patients with severe LVOTO and secondary MR who are poor surgical candidates. Mechanistically, TEER approximates the mid-leaflets, restricting the systolic mobility of the anterior leaflet. A patient-level meta-analysis of 30 patients across 15 publications confirmed a marked reduction in both resting and provocable gradients. Individual case examples reported in the literature provide useful visual representations of how clip deployment mechanically abolishes SAM and alters left atrial pressures; however, clinical decision-making regarding TEER must be driven by comprehensive Heart Team evaluation rather than isolated success stories [73,74,75].

### 9.3. Medical Management

#### 9.3.1. Traditional Pharmacological Therapy

The initial management of symptomatic HOCM is fundamentally pharmacological, aiming to attenuate left ventricular hypercontractility and optimize diastolic filling times. First-line medical therapy incorporates high-dose beta-blockers (e.g., metoprolol) or non-dihydropyridine calcium channel blockers (e.g., verapamil, diltiazem). Long-term data from surgical cohorts underscore that beta-blocker therapy should often be maintained post-myectomy; abrupt cessation can provoke symptom recurrence even in the absence of a demonstrable resting gradient. For patients who remain severely symptomatic despite maximized dual therapy, the addition of disopyramide—a Class IA antiarrhythmic possessing potent negative inotropic properties—is highly effective in suppressing dynamic gradients, though its use requires close monitoring of the QTe interval [76].

Concomitant beta-blocker therapy post-septal reduction has been associated with enhanced survival in patients requiring combined myectomy and surgical AVR. This survival benefit appears independent of direct gradient reduction, suggesting intrinsic protective myocardial effects against arrhythmias and remodeling. Despite optimized traditional medical therapy, approximately 37% of patients across major cohorts continue to report persistent, life-altering symptoms, highlighting the limitations of conventional agents [77].

#### 9.3.2. Cardiac Myosin Inhibitors: Mavacamten and Future Agents

Mavacamten, a first-in-class selective cardiac myosin inhibitor, represents a significant re-alignment of clinical practice in the medical management of obstructive HCM by targeting the underlying pathophysiology at the molecular level. By specifically inhibiting cardiac myosin ATPase, mavacamten reduces excessive actin-myosin cross-bridge formation. This decreases sarcomere-level hypercontractility, alleviates myocardial hyperkinesis, and suppresses SAM-induced LVOTO without compromising systemic cardiac output [78].

The pivotal VALOR-HCM randomized, placebo-controlled trial demonstrated the profound clinical efficacy of mavacamten in symptomatic obstructive HCM patients. Crucially, only 17.9% of mavacamten-treated patients met standardized clinical guidelines for septal reduction therapy (SRT) at follow-up compared to 76.8% in the placebo arm, representing an absolute risk reduction of 58.9% (*p* < 0.001). Patients randomized to mavacamten achieved a mean reduction in post-exercise peak LVOT gradient of 37.2 mmHg and a significant optimization of NYHA class. Biomarker analysis revealed a geometric reduction in N-terminal pro-B-type natriuretic peptide (NT-proBNP) and cardiac troponin I, establishing mavacamten as a powerful pharmacological alternative that can safely defer or eliminate the need for invasive septal reduction [79,80].

Real-world clinical data from the multicenter MARVEL-HCM registry confirmed the durability of mavacamten monotherapy. Among patients managed with mavacamten monotherapy, 100% achieved a resting gradient ≤ 30 mmHg and improved to NYHA functional class I/II at 60 weeks, with half the cohort achieving complete symptom eradication (NYHA Class I). The therapy demonstrated a favorable safety profile, with a modest, fully reversible mean decline in left ventricular ejection fraction (LVEF) of only 4.8%; temporary dose adjustments were required in only one patient whose LVEF dropped below 50% [81].

The unique mechanism of mavacamten offers significant theoretical advantages over traditional negative inotropes, which often fail to optimize systemic perfusion. By modulating sarcomere energetics, mavacamten addresses the core molecular driver of HCM. Long-term surveillance trials are ongoing to determine if early initiation can halt progression toward heart failure with reduced ejection fraction or attenuate the burden of sudden cardiac death [82].

Mavacamten has also shown utility in complex post-procedural scenarios. For example, patients developing severe, recurrent MR and dynamic LVOTO post-TAVR represent a high-risk group where standard negative inotropes may be contraindicated due to systemic hypotension. In an illustrative case of an 83-year-old male presenting with post-TAVR obstruction, initiation of mavacamten achieved complete resolution of the outflow tract gradient (>100 mmHg to normal) within two months, down-staging MR to mild while preserving normal prosthetic valve function [78].

While mavacamten achieves notable reductions in left ventricular outflow tract gradients and functional improvements, its therapeutic window requires strict clinical oversight:

- Mandatory Echocardiographic Surveillance: Due to its direct mechanism of action—inhibiting cardiac myosin ATPase to attenuate hypercontractility—mavacamten carries an inherent risk of causing systolic dysfunction. Safe deployment relies on routine echocardiographic monitoring to track left ventricular ejection fraction (LVEF) before initiation, during dose titration, and at established maintenance intervals.
- Transient LVEF Reduction: In clinical cohorts (including real-world evaluations like the MARVEL registry), mavacamten therapy is associated with modest, typically reversible declines in LVEF. In instances where LVEF drops below safety thresholds (e.g., <50%), protocolized dose reductions or temporary drug interruptions are required until baseline systolic function recovers.
- Pharmacokinetics & Drug Interactions: Mavacamten is extensively metabolized by hepatic cytochrome P450 enzymes (primarily CYP2C19 and CYP3A4). Co-administration with strong/moderate CYP inhibitors or inducers can significantly alter drug exposure, elevating the risk of severe LV dysfunction or rendering the treatment ineffective. Consequently, strict drug-interaction screening is essential prior to initiation.

Because mavacamten acts downstream on the sarcomeric contractile apparatus without permanently altering underlying septal or valvular geometry, treatment is chronic. Cessation of therapy typically leads to a rebound of hypercontractility, recurrence of dynamic gradients, and returning symptoms.

As a novel target-specific biologic agent, the financial burden of chronic mavacamten therapy is substantial compared to inexpensive traditional pharmacotherapy (e.g., non-selective beta-blockers, verapamil, or disopyramide). Financial barriers and insurance coverage logistics represent critical non-clinical parameters influencing patient compliance and real-world adoption [83].

To optimize pharmacokinetics and minimize off-target effects, second-generation cardiac myosin inhibitors (such as aficamten) are actively undergoing advanced clinical translation.

Clinical evaluations, including the pivotal SEQUOIA-HCM trial, demonstrate that aficamten achieves significant reductions in resting and provoked LVOT gradients, marked functional improvements in NYHA class, and gains in peak oxygen consumption. Crucially, aficamten possesses a shorter terminal half-life (approx. 3.4 days) and a wider therapeutic index compared to mavacamten, enabling a predictable, rapid dose-titration scheme (achieving steady-state within weeks rather than months). Furthermore, it exhibits minimal dependence on cytochrome P450 pathways, markedly reducing the risk of major drug–drug interactions. While routine echocardiographic monitoring remains essential to track left ventricular ejection fraction (LVEF), aficamten’s favorable pharmacokinetic profile provides a promising, highly manageable therapeutic alternative to invasive septal reduction strategies [84]. Concurrently, novel metabolic modulators like SGLT2 inhibitors are being evaluated for their ability to enhance myocardial energetics. Looking forward, gene-editing therapies targeting specific sarcomeric mutations hold promise for delivering definitive therapeutic options [82].

The role of mavacamten within the therapeutic algorithm for oHCM continues to evolve. According to current guidelines (e.g., 2024 AHA/ACC HCM Guidelines), cardiac myosin inhibitors represent a valuable pharmacological option for adult patients with symptomatic oHCM (NYHA class II–III) who remain symptomatic despite maximally tolerated first-line therapy (beta-blockers or non-dihydropyridine calcium channel blockers). While clinical trial protocols (such as VALOR-HCM) evaluated mavacamten as a strategy to defer or avoid septal reduction therapy (SRT), guidelines do not dictate a fixed mandatory duration (such as 4 to 12 weeks) before SRT referral. Instead, the decision to initiate mavacamten or proceed to invasive SRT should be tailored within a multidisciplinary Heart Team framework, considering patient candidacy, preference, eligibility for REMS monitoring, and local expert surgical availability [50,78].

## 10. Clinical Outcomes and Complications

### 10.1. Short-Term Outcomes and In-Hospital Complications

Surgical septal myectomy achieves low in-hospital mortality when performed at expert centers. In a benchmark series of 84 patients undergoing extended myectomy, the long-term 12-year survival rate was excellent at 78 ± 22%. The acute in-hospital requirement for permanent pacemaker implantation due to complete atrioventricular block was 5%, with the cumulative incidence rising to 15 ± 7% during long-term follow-up [55].

A comprehensive analysis comparing myectomy against ASA utilizing the National Inpatient Sample (2011–2019) revealed distinct complication profiles. Patients undergoing surgical myectomy demonstrated higher adjusted odds of perioperative all-cause mortality (OR 1.8), ischemic stroke (OR 2.3), acute kidney injury (OR 1.4), major vascular complications (OR 3.6), ventricular septal defects (OR 4.4), and new-onset left bundle branch block (LBBB, OR 3.5). Conversely, patients undergoing percutaneous ASA experienced a significantly higher incidence of complete heart block (OR 1.3), new right bundle branch block (RBBB, OR 6.3), peri-procedural ventricular tachycardia (OR 2.2), and permanent pacemaker dependency (OR 6.4) [85].

### 10.2. Ventricular Arrhythmias and Sudden Cardiac Death Risk

The localized myocardial scar induced by alcohol septal ablation can serve as a proarrhythmic substrate. A major meta-analysis encompassing 20 studies and 8025 patients identified a significant discrepancy in arrhythmic burdens. Sustained ventricular tachycardia or ventricular fibrillation (VT/VF) occurred in 10% of the ASA cohort compared to 5% of the surgical myectomy cohort (RR 1.98, *p* < 0.00001). While the majority of these events manifested during the acute procedural phase or index hospitalization, the elevated risk of ventricular arrhythmias persisted into the late follow-up phase (RR 2.80, *p* = 0.055). Crucially, the long-term risk of sudden cardiac arrest was significantly higher in patients managed with ASA compared to surgery (RR 2.30, *p* = 0.002) [86].

### 10.3. Pacemaker Implantation

High-grade atrioventricular block requiring permanent pacemaker deployment represents a major complication of all septal reduction therapies. Data from the Japanese nationwide registry of 634 ASA patients documented a baseline high-grade heart block rate of 7.9%, identifying female sex as an independent predictor of conduction system injury. In a separate cohort of 30 mildly symptomatic HOCM patients undergoing early elective ASA, the permanent pacemaker implantation rate was 10%, which was considered acceptable when compared to historical data [87,88].

### 10.4. Long-Term Survival and Quality of Life

Long-term survival following appropriate referral for septal reduction therapy is outstanding. In a longitudinal cohort of 477 HOCM patients referred to a tertiary center, combined procedural mortality for both myectomy and repeat ASA was 0.3%. Functional optimization to NYHA class I/II was sustained in 95% of survivors, and long-term survival was statistically equivalent to age- and sex-matched general populations [89].

An analysis of the National Readmission Database (2010–2019) comprising 19,007 encounters emphasized a marked volume-outcome relationship for surgical myectomy. High-volume centers (>28 myectomies/year) demonstrated significantly lower mortality rates compared to low-volume centers (<8 myectomies/year). In contrast, post-procedural mortality following percutaneous ASA remained independent of institutional volume, suggesting that the percutaneous technique may offer more consistent reproducibility across diverse clinical settings [90].

## 11. Special Populations and Challenging Cases

### 11.1. SAM in Cardiac Amyloidosis

Systolic anterior motion can occasionally manifest in infiltrative disorders such as cardiac amyloidosis. Documented cases describe patients with amyloid light-chain (AL) or transthyretin (ATTR) amyloidosis who present with thickened leaflets and develop profound exertional syncope. Exercise stress echocardiography in these individuals unmasks severe SAM and a dynamic late-peaking LVOT gradient driven by exertional hypercontractility of non-infiltrated basal segments. Consequently, a comprehensive echocardiographic evaluation is mandatory in amyloid patients presenting with syncope prior to empirical permanent pacemaker implantation [91].

### 11.2. SAM in Fabry Disease

In contrast to HOCM, where the prevalence of SAM ranges from 25% to 50%, SAM is exceedingly rare in Fabry disease despite the presence of severe concentric left ventricular hypertrophy and papillary muscle enlargement. In a contemporary series of 42 patients with confirmed Fabry disease and moderate-to-severe MR, zero instances of SAM were documented, highlighting a distinct pathophysiological mechanism of valve interaction in lysosomal storage disorders [92].

Dynamic hemodynamic fluid shifts can induce transient SAM in patients with baseline concentric hypertrophy. For example, a 37-year-old dialysis patient with known LVH developed severe, refractory LVOTO, SAM, and acute hemodynamic collapse six months following the surgical reopening of a high-flow arteriovenous fistula (AVF). Meticulous surgical ligation of the AVF reduced the venous return and hyperkinetic state, achieving complete resolution of SAM. This underscores the necessity of rigorous cardiovascular risk stratification prior to establishing high-flow vascular access in patients with pre-existing structural heart disease [93].

## 12. Emerging Therapeutic Approaches

### 12.1. Novel Ablation Techniques

Percutaneous intramyocardial septal radiofrequency ablation (PIMSRA) has emerged as a novel, ultrasound-guided alternative to alcohol-based ablation. However, atypical complications can manifest; cases of acute proximal displacement of the anterior leaflet inducing hemodynamic shock have been documented one hour post-PIMSRA, though complete spontaneous recovery was achieved within one week. A randomized trial comparing percutaneous endocardial septal radiofrequency ablation (PESA) against traditional ASA in 80 patients confirmed that while both techniques significantly attenuated gradients, ASA achieved superior reduction in septal thickness and NYHA class. Crucially, however, patients managed with PESA demonstrated a significantly lower incidence of post-procedural bundle branch blocks [93,94,95].

Alternative chemical agents have been evaluated to refine percutaneous ablation. Sclerotherapy utilizing polidocanol achieved significant gradient reduction (76.5 to 30 mmHg) in a single-center series of 28 patients, though it carried a 14.3% permanent pacemaker requirement and a 64.2% rate of new conduction defects. Similarly, particulate embolic agents have been deployed to minimize the systemic risk of ethanol leakage, though long-term data suggest an increased risk of future target-lesion re-intervention [96,97].

### 12.2. Dual-Chamber Pacemaker Therapy

In HOCM patients who present with prohibitive coronary anatomy that precludes both surgical myectomy and percutaneous ASA, dual-chamber (DDD) pacing represents a viable alternative. While individual case descriptions vividly illustrate how programming a short atrioventricular delay alters the systolic contraction vector to decrease peak gradients, indication for pacing must remain grounded in established consensus guidelines for refractory, highly selected cases rather than isolated anecdotal reports [98].

## 13. Comparison of Treatment Strategies

### 13.1. Direct Comparative Analyses

A nationwide French retrospective cohort study utilizing the PMSI database (2010–2019) propensity-matched 340 surgical myectomy patients with 1234 ASA patients. At mid-term follow-up, all-cause mortality was statistically identical between the cohorts. However, the risk of all-cause ischemic stroke was significantly lower in the percutaneous ASA group (adjusted IRR 0.180, *p* = 0.003). Interestingly, despite European Society of Cardiology (ESC) guidelines favoring surgical myectomy at expert centers, percutaneous ASA was more frequently performed in real-world clinical practice [99].

Head-to-head functional data confirm that surgical myectomy achieves superior long-term gradient suppression compared to ASA (mean post-procedural gradient 21.0 mmHg vs. 34.3 mmHg, *p* < 0.001). Furthermore, a significantly higher percentage of surgical patients achieve complete symptom eradication to NYHA functional class I (80.4% vs. 48%), and myectomy enables more reliable discontinuation of negative inotropic medications such as disopyramide [56].

### 13.2. Outcomes Following Prior Septal Reduction Therapy

Surgical myectomy serves as a safe and effective rescue intervention for patients presenting with recurrent or residual LVOTO following a failed prior percutaneous ablation. In a comparative series of 145 patients undergoing re-intervention myectomy (72 with prior ASA, 73 with a failed prior myectomy), the incidence of new complete heart block requiring pacemaker implantation was statistically comparable between the groups (8.3% vs. 2.7%, *p* = 0.151). Long-term 15-year survival rates remained excellent and did not differ significantly between the post-ASA rescue group and the primary re-myectomy group (76% vs. 64%, *p* = 0.207) [100].

## 14. The Multidisciplinary Heart Team Approach

The complexity and heterogeneity of this disease presentation necessitate systematic multidisciplinary evaluation involving cardiology (general, imaging, electrophysiology expertise), cardiac surgery, clinical genetics/genetic counseling, and allied healthcare professionals. Multidisciplinary team assessment ensures comprehensive diagnostic evaluation including consideration of disease phenotype, genetic basis, imaging findings across modalities, arrhythmic risk profile, and functional capacity. This integrated approach enables evidence-based selection among diverse therapeutic options ranging from conservative medical management with beta-blockers or calcium channel blockers, through pharmacological innovations including cardiac myosin inhibitors (mavacamten and aficamten), to invasive septal reduction therapies (surgical myectomy or alcohol septal ablation) and device-based interventions. Shared decision-making frameworks explicitly incorporating patient values, preferences regarding symptom burden and quality of life, and individual risk-benefit assessments of available interventions represent best practice in HCM management. The multidisciplinary approach particularly benefits complex cases involving rare anatomical variants, phenocopies, comorbid conditions, pregnancy planning, and exercise participation counseling [101].

**Figure 1:**
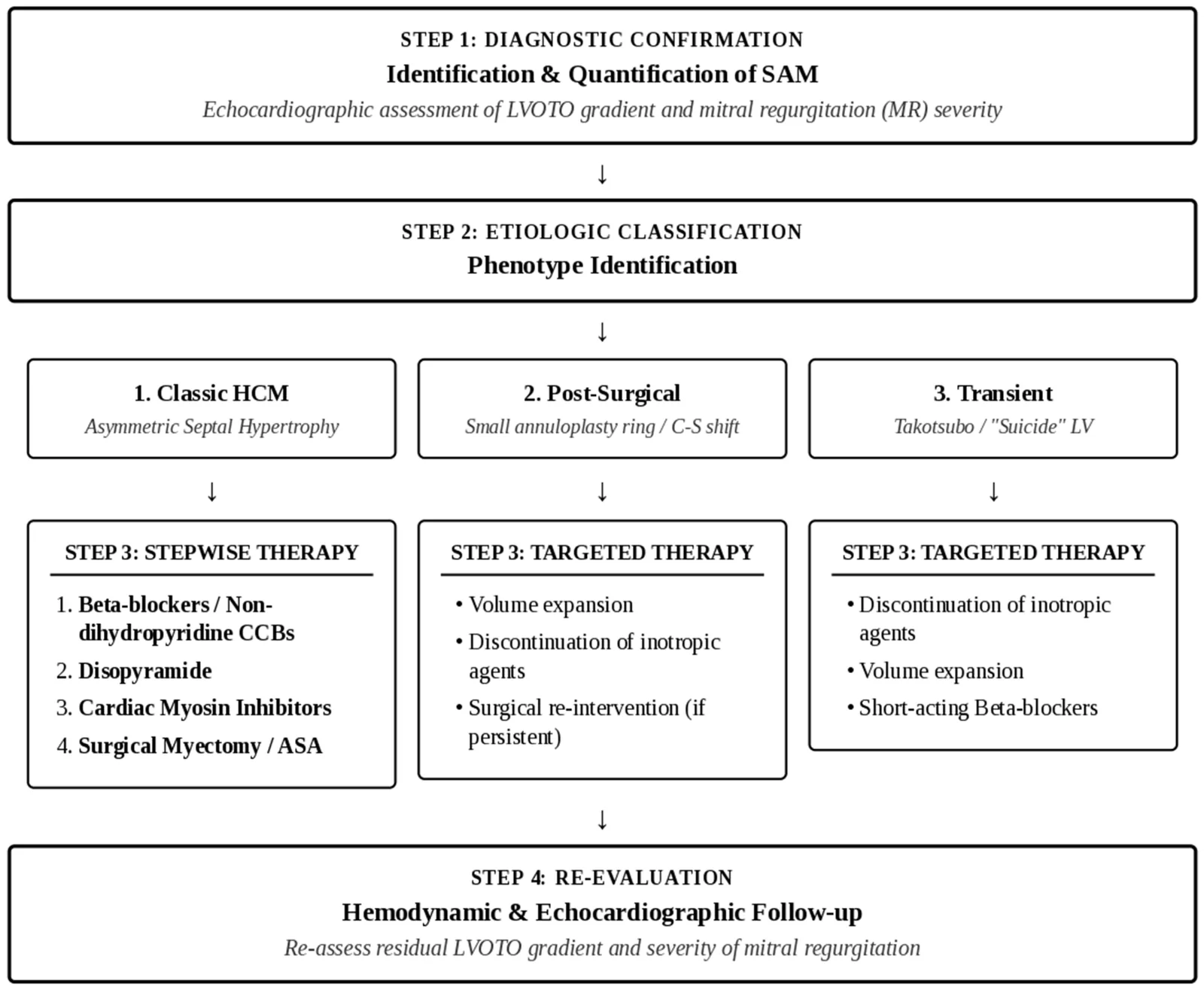
Management Algorithm for Systolic Anterior Motion (SAM) of the Mitral Valve. Proposed clinical workflow for the evaluation and management of SAM based on underlying etiology, structural findings, and hemodynamic presentation [50,52,53,54,55,64,69,70,72,73,74,76,78,79,80]. Abbreviations: ASA, Alcohol Septal Ablation; CCB, Calcium Channel Blocker; C-S, Coaptation-to-Septum distance; HCM, Hypertrophic Cardiomyopathy; LV, Left Ventricle; LVOTO, Left Ventricular Outflow Tract Obstruction; MR, Mitral Regurgitation; SAM, Systolic Anterior Motion.

Management of systolic anterior motion of the mitral valve varies depending on the underlying etiologic phenotype, as outlined in the management algorithm (Figure 1).

Contemporary evidence demonstrates substantial volume-outcome relationships for septal reduction therapies (myectomy and alcohol septal ablation), underscoring the critical importance of referral to experienced high-volume centers. Analysis of 5725 HCM patients (3990 septal myectomy, 1735 ASA) from the Vizient Clinical Data Base (2016–2022) stratified by annualized operator and hospital volumes into low-, medium-, and high-volume groups revealed striking outcome differences. For septal myectomy, low-volume operators demonstrated substantially higher odds of 30-day mortality (adjusted odds ratio 1.86, 95% CI 1.11–3.15) and greater 90-day readmission risk (adjusted odds ratio 1.51, 95% CI 1.22–1.88); medium-volume operators also showed increased 30-day mortality (adjusted odds ratio 1.93, 95% CI 1.05–3.55). Medium-volume hospitals exhibited higher 30-day mortality (adjusted odds ratio 2.29, 95% CI 1.32–3.99), while low-volume hospitals demonstrated greater 90-day readmission risk (adjusted odds ratio 1.60, 95% CI 1.14–2.23). For alcohol septal ablation, low- and medium-volume operators demonstrated increased 30-day mortality (respective adjusted odds ratios 2.99 and 3.86), though hospital volume did not significantly impact ASA outcomes [102]. These compelling data establish volume-outcome relationships as a critical driver of procedural outcomes and provide evidence-based justification for systematic referral of HCM patients requiring interventional therapy to centers of excellence with experienced operators and infrastructure.

Despite accumulating evidence supporting comprehensive specialized evaluation and treatment in dedicated HCM centers, most HCM patients continue to receive care in non-specialized settings with limited access to advanced diagnostics and interventions [103].

Substantial regional variations in HCM diagnosis, investigation, and treatment pathways reflect disparity in access to specialized expertise and multimodal imaging. In a 14-year cohort analysis of 8514 German HCM patients undergoing septal reduction therapy (5293 ASA, 3221 myectomy) between 2006 and 2019, the majority of procedures were performed at hospitals in the lowest volume category (≤20 procedures) [34]. Low myectomy volume at hospitals was independently associated with increased risk of in-hospital mortality (adjusted odds ratio 2.64, 95% CI 1.49–4.66 comparing lowest to highest tertile), whereas low alcohol septal ablation volume was not independently associated with adverse outcomes [104].

## 15. Conclusions

Systolic anterior motion of the mitral valve is a dynamic, structurally diverse phenomenon affecting a heterogeneous spectrum of patient populations, ranging from classical HOCM to post-operative iatrogenic repairs, congenital lesions, and rare metabolic conditions like Fabry disease. Innovations in three-dimensional transesophageal echocardiography have significantly advanced the spatial characterization of the subvalvular apparatus. Clinicians must recognize that SAM is not exclusive to increased septal thickness; dynamic fluid shifts and intrinsic geometric leaflet abnormalities play equal roles in driving obstruction.

Surgical myectomy remains the established therapeutic standard for drug-refractory symptomatic HOCM. When performed at high-volume centers, surgical mortality is low and long-term survival is outstanding. In patients presenting with co-existing structural mitral valve anomalies, selective and anatomy-dependent interventions such as the Alfieri edge-to-edge repair or papillary muscle reorientation should be deployed to ensure procedural durability. For individuals deemed at prohibitive surgical risk and possessing favorable anatomical criteria percutaneous options like ASA or TEER may offer selective alternatives though they carry higher long-term risks of pacemaker dependency, re-intervention, or ventricular arrhythmias. The introduction of selective cardiac myosin inhibitors like mavacamten marks a major advance, providing a targeted, non-invasive option to modify the disease at the molecular level.

Managing non-hypertrophic SAM depends on identifying the specific trigger. Post-operative SAM following mitral repair can frequently be stabilized with aggressive fluid and beta-blocker titration, while surgical re-intervention should focus on restoring proper valve geometry. Ultimately, optimizing outcomes across all SAM phenotypes requires an experienced, multidisciplinary Heart Team utilizing advanced multi-modality imaging to deliver tailored care aligned with patient values.

## Figures and Tables

**Table 1:** **Main phenotypes of SAM:** Summary of the distinct clinical SAM phenotypes, outlining their defining anatomical characteristics alongside the core physiological and cellular mechanisms driving each condition [1,4,6,7,8,9,10,11,12,13,14,15,16,17,18,19,20,21,22,23,24,25,26,27,28,29,30,31].

SAM Phenotype	Key Anatomical Features	Primary Mechanisms
HCM-related SAM	Septal hypertrophy, elongated anterior leaflet, anterior papillary muscle displacement	Drag forces, Venturi effect, diastolic anterior motion
Post-MVR SAM	Altered annular geometry, anteriorly positioned coaptation point, annuloplasty ring effect	Geometric reconfiguration, shortened C-Sept distance
Post-AVR/TAVR SAM	Short mitral-aortic annular angle, concentric hypertrophy, normal or small cavity	Pressure relief paradox, negative transmitral gradients
Transient SAM	No structural abnormality; apical dysfunction with basal hyperkinesis	Flow acceleration, negative pressure gradients, catecholamine effects
Congenital/anatomic SAM	Isolated leaflet elongation, accessory tissue, abnormal septal geometry without HCM phenotype	Direct leaflet–septal contact from elongation or anatomic abnormality
Midventricular obstruction	Papillary muscle hypertrophy, no leaflet–septal contact, circumferential obstruction	Papillary muscle-mediated obstruction, increased apical wall stress

## Data Availability

No new data were created or analyzed in this study.

## Abbreviations

The following abbreviations are used in this manuscript:

Abbreviation	Full Term
SAM	Systolic Anterior Motion
LVOT	Left Ventricular Outflow Tract
MR	Mitral Regurgitation
HOCM	Hypertrophic Obstructive Cardiomyopathy
LVOTO	Left Ventricular Outflow Tract Obstruction
LVH	Left Ventricular Hypertrophy
3D-TEE	Three-Dimensional Transesophageal Echocardiography
CLI	Cleft-Like Indentation
CCHR	Complete Clinical and Hemodynamic Response
TEER	Transcatheter Edge-to-Edge Repair
ASA	Alcohol Septal Ablation
AVR	Aortic Valve Replacement
pAVSD	Partial Atrioventricular Septal Defect
TAVR	Transcatheter Aortic Valve Replacement
AV	Atrioventricular
PIMSRA	Percutaneous Intramyocardial Septal Radiofrequency Ablation
PESA	Percutaneous Endocardial Septal Radiofrequency Ablation
AVF	Arteriovenous Fistula
NYHA	New York Heart Association
OR	Odds Ratio
CI	Confidence Interval
RR	Risk Ratio
SM	Surgical Myectomy
TEE	Transesophageal Echocardiography
ICD	Implantable Cardioverter Defibrillator
